# Reporting of sex and gender in randomized controlled trials in Canada: a cross-sectional methods study

**DOI:** 10.1186/s41073-017-0039-6

**Published:** 2017-09-01

**Authors:** V. Welch, M. Doull, M. Yoganathan, J. Jull, M. Boscoe, S. E. Coen, Z. Marshall, J. Pardo Pardo, A. Pederson, J. Petkovic, L. Puil, L. Quinlan, B. Shea, T. Rader, V. Runnels, S. Tudiver

**Affiliations:** 1 Bruyère Research Institute, Bruyère Continuing Care, 304b-85 Primrose Avenue, Ottawa, Ontario K1R 6 M1 Canada; 2 University of Ottawa, Ontario, Canada; 3 School of Nursing, University of British Columbia, T223-2211 Wesbrook Mall, Vancouver, British Columbia V6T 2B5 Canada; 4 Ottawa Hospital Research Institute, University of Ottawa, Ottawa, Ontario Canada; 5 Research Sex/gender, Health Equity, Primary Care Consultant, 906 Bowron Court, North Vancouver, BC V7H 2S7 Canada; 6 Department of Geography and Planning, Queen’s University, Mackintosh-Corry Hall, Kingston, Ontario K7L 3 N6 Canada; 7 Renison University College, University of Waterloo, 240 Westmount Road North, Waterloo, Ontario N2L 3G4 Canada; 8 Cochrane Musculoskeletal, University of Ottawa, Ottawa Hospital Research Institute, The Ottawa Hospital, General Campus, 501 Smyth Road, Ottawa, ON K1H 8L6 Canada; 9 B.C. Women’s Hospital + Health Centre, E305, 4500 Oak Street, Vancouver, BC V6H 3E1 Canada; 10 Department of Anesthesiology, Pharmacology & Therapeutics, Faculty of Medicine, University of British Columbia, 2176 Health Sciences Mall, Vancouver, BC V6T 1Z3 Canada; 11 Canadian Agency for Drugs and Technology in Health, 865 Carling Ave, Ottawa, Ontario Canada; 12 Globalization and Health Equity Research Unit, School of Epidemiology, Public Health and Preventive Medicine, University of Ottawa, 850 Peter Morand Crescent, Ottawa, Ontario K1G 5Z3 Canada; 13 Gender and Health Consultant, 161 Northwestern Avenue, Ottawa, Ontario K1Y 0 M1 Canada

**Keywords:** Sex/gender analysis, Gender, Randomized controlled trials as a topic, Canada, Quality

## Abstract

**Background:**

Accurate reporting on sex and gender in health research is integral to ensuring that health interventions are safe and effective. In Canada and internationally, governments, research organizations, journal editors, and health agencies have called for more inclusive research, provision of sex-disaggregated data, and the integration of sex and gender analysis throughout the research process. Sex and gender analysis is generally defined as an approach for considering how and why different subpopulations (e.g., of diverse genders, ages, and social locations) may experience health conditions and interventions in different or similar ways.

The objective of this study was to assess the extent and nature of reporting about sex and/or gender, including whether sex and gender analysis (SGA) was carried out in a sample of Canadian randomized controlled trials (RCTs) with human participants.

**Methods:**

We searched MEDLINE from 01 January 2013 to 23 July 2014 using a validated filter for identification of RCTs, combined with terms related to Canada. Two reviewers screened the search results to identify the first 100 RCTs that were either identified in the trial publication as funded by a Canadian organization or which had a first or last author based in Canada. Data were independently extracted by two people from 10% of the RCTs during an initial training period; once agreement was reached on this sample, the remainder of the data extraction was completed by one person and verified by a second.

**Results:**

The search yielded 1433 records. We screened 256 records to identify 100 RCTs which met our eligibility criteria. The median sample size of the RCTs was 107 participants (range 12–6085). While 98% of studies described the demographic composition of their participants by sex, only 6% conducted a subgroup analysis across sex and 4% reported sex-disaggregated data. No article defined “sex” and/or “gender.” No publication carried out a comprehensive sex and gender analysis.

**Conclusions:**

Findings highlight poor uptake of sex and gender considerations in the Canadian RCT context and underscore the need for better articulated guidance on sex and gender analysis to improve reporting of evidence, inform policy development, and guide future research.

**Electronic supplementary material:**

The online version of this article (doi:10.1186/s41073-017-0039-6) contains supplementary material, which is available to authorized users.

## Background

Over the past several decades, there has been increasing awareness within the community of health researchers, funders, and knowledge users (e.g., policy-makers, practitioners, and patients) of the need to understand how sex and gender influence health outcomes [1, 2]. “Sex” is commonly used to refer to genetic, biological, and physiological processes; whereas “gender” is commonly used to refer to the roles, relationships, behaviors, relative power, and other traits that societies ascribe to women, men, and people of diverse gender identities [3]. Sex and gender interact with each other and other characteristics to influence health outcomes [4–9]. For example, research indicates there are significant physiological differences in cardiac function between males and females such as susceptibility to QT interval prolongation and serious heart arrhythmias as well as gender differences in how men and women who have heart disease are diagnosed and treated [10–12]. Failure to take these differences into account, not just between men and women, but also across other characteristics such as age and socioeconomic status, can have serious, even life-threatening, consequences for individual patients.

As used in this paper, sex and gender analysis is an approach and framework for considering fundamental questions about how and why different subpopulations (e.g., of diverse genders, ages, and social locations) may experience health conditions and interventions in different or similar ways. These fundamental questions are systematically applied to all stages of the research process, starting with the formulation of the initial research question, followed by the development of methodology, conduct of the analysis, and interpretation of results and reflecting on their implications [13–16].

Policies and guidance increasingly mandate or recommend routine collection, reporting and analysis of the influence of sex and gender in scientific research. However, uptake of sex and gender analysis, and its impacts on reporting and on health outcomes remain unclear [2, 17, 18]. For example, funding policies of the U.S. National Institutes of Health (Revitalization Act 1993) [19], mandating the appropriate inclusion of women and minorities in clinical trials have resulted in increased inclusion of diverse populations in some areas of health research [20, 21] but not all [22]. Furthermore, sex and gender analysis to assess similarities or differences in health outcomes remains limited [23]. In the Canadian federal context, neither Health Canada’s 1997 Guidance Document on Inclusion of Women in Clinical Trials nor the more detailed May 2013 document that replaced it, “Considerations for Inclusion of Women in Clinical Trials and Analysis of Sex Differences” [24], provide mechanisms to track implementation of this guidance by researchers and sponsors of clinical trials in Canada to identify outcomes in relation to sex and gender considerations. In 2011, the Canadian Institutes of Health Research (CIHR) implemented a requirement that all research grant applicants indicate whether their research proposal addresses sex and gender and to provide justification for their response [25, 26]. The preliminary results assessing the implementation of this policy indicate increased consideration of sex and gender in most categories of research proposals [26], but the impact of the policy on the conduct and reporting of research, including randomized controlled trials (RCTs), has yet to be examined.

The objective of this study was to provide a preliminary assessment of the extent and nature of reporting about sex and/or gender, including whether sex and gender analysis was carried out, in a sample of recently published Canadian RCTs with human participants.

## Methods

A collaborative research team (all authors on this paper) engaged in a deliberative, consensus building process and team meetings conducted to plan, develop, and conduct a cross-sectional study to meet our objective.

Consensus was reached on a data extraction form and methods for the study. Our working definitions of sex and gender were adapted from the Canadian Institutes of Health Research, Canada’s health research funding agency, which recognizes sex and gender as analytically distinct but interdependent concepts and which also acknowledges the nuances of sex and gender beyond the binary [27].

For the purposes of our search, Canadian trials were defined as those that were either identified in the publication as funded by a Canada-based funder and/or had a first or last author with affiliation based in Canada. To account for the diverse nature of Canadian trials, which can involve cross-border and inter-sectoral collaborations, we included multi-centre trials, as well as trials funded jointly by Canadian and international sources. We did not restrict inclusion on the basis of age (e.g., children, adolescents) or whether the trial focused on specific populations (including across sex and/or gender). We chose a sample size of 100 RCTs based on the sample size of other similar methodological studies [23, 28, 29]. This sample size of 100 trials will detect a proportion of 25% with a 95% confidence interval of ±8.5%.

A search strategy was developed in consultation with a librarian scientist (TR) to identify Canadian RCTs conducted with human participants. This strategy comprised a MEDLINE search using the OVID interface with the specific filter (randomized controlled trial.pt OR randomized controlled trial.mp) combined with Canadian provinces/territories (Quebec or Ontario or “Prince Edward Island” or New Brunswick or Nunavut or “Northwest Territories” or Nova Scotia or Newfoundland or Labrador or Yukon or British Columbia or Manitoba or Alberta or Saskatchewan).in OR (Canadian or Canada).in) from 01 January 2013 to 23 July 2014. We chose to search MEDLINE up to 1 year prior to data collection because most articles would be indexed according to Medical Subject Headings by this time, allowing the use of the specific filter for randomized trials.

Two authors screened records independently (duplicate screening) in order of date of publication, starting with the most recent, to identify those RCTs meeting eligibility criteria.

A data extraction form was developed and pre-tested to capture data on the type of intervention, study design (cluster or individually randomized trial), sample size, and funding sources. We assessed whether sex and gender analysis was conducted, what was done and how this was reported, drawing from items in the PRISMA-Equity extension [30] and the European Association of Science Editors (EASE) guidance [31]. Details on whether sex or gender were mentioned and in what context were collected from the title/abstract, introduction, methods, eligibility criteria, population characteristics, results, subgroup analysis, interpretation of applicability, and discussion. We also collected data on reporting of social determinants of health (e.g., socioeconomic status, occupation, place of residence) which will be reported in a separate paper (contact first author for details) (see Additional file 1: Appendix 1 for complete list of data extraction items).

Data were independently extracted by two people from 10% of the RCTs as a training exercise: once agreement was reached on this sample [32] the remainder of the data extraction was completed by one person (one of TB, LQ, and MY) and was verified by a second (one of JJ, VW).

We included both primary publications of RCTs as well as secondary publications (i.e., secondary analyses on RCTs that had already been published). For 13 secondary publications, we identified the primary RCT publication and used that as the basis for the data extraction, and supplemented with information from the secondary publication. Secondary publications were included because subgroup analyses are sometimes reported in secondary, follow-up publications rather than in the primary trial report.

## Results

### Search results

**Fig. 1:**
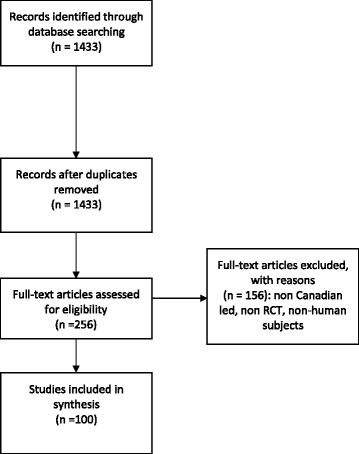
Study flow chart

The search identified 1433 records. We screened 256 records from the most recent to the oldest to identify the first 100 eligible RCTs. Of the ineligible records, 120 were excluded because they were not RCTs or involved non-human subjects. We also excluded 36 RCTs that did not have a Canadian funder or a first or last author based in Canada (Fig. 1).

### Description of the sample

#### Inclusion criteria

Canadian funding was reported in 68/100 of the RCTs (68%), while the remaining 32 trials were eligible for inclusion in the sample on the basis of having a first or last author based in Canada. Of these 32 trials, 13 were funded by non-Canadian sources and 19 did not report on any sources of funding. Of the 81 RCTs that did report on funding sources, 37 identified non-profit sources of funding; 22 identified government sources (e.g., Canadian Institutes of Health Research, Health Canada); 10 indicated they were solely funded by industry; while 8 RCTs identified a combination of non-profit and government sources; 2 identified non-profit and industry funding and 2 identified government and industry sources of funding (Additional file 2: Appendix 2).

#### Population

We classified trials as “single-sex” and “mixed-sex”, based on the terminology used by Gendered Innovations [33]. Twelve RCTs were classified as single-sex studies because they had eligibility criteria that restricted participation to women, one RCT enrolled girls and one RCT enrolled boys. The remainder of RCTs were classified as mixed-sex because they enrolled male and female participants. Eighty-five out of 88 mixed-sex RCTs reported the number of enrolled male and female participants. For these, the median number of male participants was 45 (range 3–3843) and the median number of female participants was 53 (range 1–1712). As shown in Table 1, the median sample size of the 100 RCTs was 103 (range 20–1466) for single sex studies and 107 participants (range 12–6085) for mixed-sex studies. As noted, we did not restrict inclusion on the basis of age or focus on specific populations. Two trials did not provide any information about the sex of the population, referring to the population as patients or nurses/care aides.

**Table 1:** Characteristics of RCTs (*n* = 100)

Study characteristics		Single-sex RCTs (*n* = 12)	Mixed-sex RCTs (*n* = 88)	Total (100)
		*N* (%) unless otherwise specified	*N* (%) unless otherwise specified	*N* (%) unless otherwise specified
First author Canadian		11	88	99 (99%)
Last author Canadian		12	81	93 (93%)
Sample size (randomized)		103 (median) Range (20–1466)	107 (median) Range (12–6085)	107 (median) range (12–6085)
Reports recruitment methods		7 (58%)	43 (49%)	50 (50%)
Cluster RCTs		1 (8%)	8 (9%)	9 (9%)
Type of intervention	Pharmacological	5 (42%)	32 (36%)	37 (37%)
	Non-pharmacological	7 (58%)	45 (51%)	52 (52%)
	Surgical	0 (0%)	6 (7%)	6 (6%)
	Organizational	0 (0%)	5 (6%)	5 (5%)
Multi-site	Within Canada	3 (25%)	24 (27%)	27 (27%)
	Outside of Canada (includes Canada)	0 (0%)	13 (15%)	13 (13%)
	Outside of Canada (not including Canada)	0 (0%)	1 (1%)	1 (1%)
	Region NR^a^	0 (0%)	1 (1%)	1 (1%)
Single-site	Within Canada	8 (67%)	47 (53%)	55 (55%)
	Outside of Canada	1 (8%)	1 (1%)	2 (2%)
	Region NR	0 (0%)	1 (1%)	1 (1%)
Canadian funding	Yes	9 (75%)	59 (67%)	68 (68%)
	No	0 (0%)	13 (15%)	13 (13%)
	NR	3 (25%)	16 (18%)	19 (19%)
Type of funding	Non-profit	3 (25%)	34 (39%)	37 (37%)
	Government	4 (33%)	18 (20%)	22 (22%)
	Industry	0 (0%)	10 (11%)	10 (10%)
	Non-profit + government	2 (17%)	6 (7%)	8 (8%)
	Non-profit + industry	0 (0%)	2 (2%)	2 (2%)
	Government + industry	0 (0%)	2 (2%)	2 (2%)
	Not reported	3 (25%)	16 (18%)	19 (19%)

^a^ *NR* not reported. No information was provided

#### Multi- and single site

Of the 100 RCTs, 42 were reported as multi-site and 58 were single-site RCTs. Not all trials provided information on the study site(s) (Table 1). In the subset of multi-site trials that did report information on site location, 27/42 (64%) were conducted entirely within Canada and only one out of the remaining 15 included no sites within Canada. In single-site RCTs reporting location, the majority (55/58 or 95%) were conducted in Canada, 2 RCTs were conducted in other countries but had Canadian first or last authors and one RCT did not describe site location.

#### Types of interventions

The interventions included in the sample of 100 RCTs were highly diverse. These included 52 non-pharmacological interventions, such as rehabilitation techniques, food supplements (e.g., canola oil and ginseng), and cognitive behavior interventions; 37 pharmacological interventions, (e.g., the use of simvastatin in hypertension); 6 surgical interventions, (e.g., coronary artery bypass grafting); and 5 organizational interventions (e.g., simulation-based training for laparoscopic inguinal repair) (see complete list of included studies in Additional file 3: Appendix 3).

### Reporting of sex/gender in RCTs

#### Sex and gender terminology in RCTs

The terminology used by authors to describe the participant demographic composition by “sex or gender” varied. For example, some trials used the term gender and some used sex. No RCT provided or referenced a definition of sex or gender or of sex and gender analysis. No studies reported on inclusion of gender diverse participants (e.g., transgender, gender non-binary, or other gender identities). Similarly, no studies used the term cisgender or transgender to describe the populations. For this reason, reference to males and females in this review and in the source references is assumed to refer to cisgender females and males according to Schilt and Westbrook’s (2009) definition of cisgender referring to “individuals who have a match between the gender they were assigned at birth, their bodies, and their personal identity” (p. 461) [34]. Because the RCTs varied in the use of terms related to sex and gender, we use the expression sex or gender to report the results.

#### Reporting of sex or gender in the sample of RCTs

None of the RCT authors stated that they intended to conduct sex and gender analysis, nor did any do so.

**Fig. 2:**
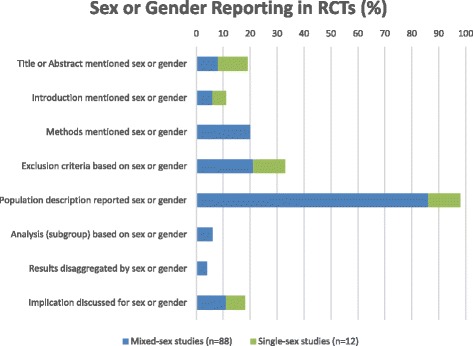
Reporting of sex and/or gender in RCTs

The extent to which sex or gender was reported across various sections of RCT publications varied considerably, as shown in Fig. 2. We provide examples of reporting to illustrate each section.

#### Title/abstract of RCTs

In the title or abstract, 19 (19%) RCTs reported on some aspect of sex or gender.

##### Single-sex studies

In the title or abstract, 11 out of 12 (92%) single-sex RCTs reported some aspect of sex or gender. For four of these, the population was defined in the title. For example, “Effect of a Novel Movement Strategy in Decreasing ACL Risk Factors in Female Adolescent Soccer Players: A Randomized Controlled Trial” [35].

##### Mixed-sex studies

Eight out of 88 (9%) RCTs reported on some aspect of sex or gender, and this was mentioned only in the abstract.

#### Background/rationale of RCTs

In the rationale or background section, sex or gender was only mentioned in 11 (11%) of the RCTs. *Single-sex studies*: 5 out of 12 single-sex RCTs (42%) reported on sex or gender in the background section. Three of the RCTs reported information on prevalence or importance of a condition in subpopulations. Two of these studies reported a rationale related to sex or gender.

##### Mixed-sex studies

Six out of 88 mixed-sex RCTs (7%) reported sex or gender information in the background section. One RCT reported information on prevalence across sex or gender. For example, one RCT stated that “…symptomatic knee OA (ed: osteoarthritis) occurs in 10% of men and 13% of women ages ˃59 years (pg.1837)” [36]. Five RCTs reported a rationale related to why the intervention might work differently across sex or gender or provided background evidence about differential effects. For example, one study of compression technologies for leg ulcer care stated in the background that, “women did more poorly according to one study, but two other studies found no significant effect of gender (pg. 1834)” [37]. Of these six studies, none report analyzing the effect of sex or gender but three RCTs discuss applicability of results with regards to sex or gender.

#### Eligibility criteria of RCTs

##### Single-sex studies

All 12 single-sex RCTS described an exclusion based on sex or gender. Two of these 12 RCTs (1 with pharmacological intervention and 1 with non-pharmacological intervention) excluded pregnant/breastfeeding women.

##### Mixed-sex studies

Twenty-one of the 88 mixed-sex RCTs (24%) described exclusion criteria based on sex or gender. Twelve RCTs excluded pregnant/breastfeeding women, 1 excluded women of child-bearing age, and 4 RCTs excluded both pregnant women and women of childbearing age. Thirteen of these 17 RCTs evaluated pharmacological interventions. For example, one pharmacological intervention using “low (50 mg/day) or high (200 mg/day) dose of Losartan” excluded “pregnant and lactating women”(pg. 590) [38] and another using a “supplement with a high-dose micronutrient, mineral and antioxidant preparation (K-PAX UltraH) or an identically appearing 100% RDA preparation of multivitamins and minerals” had exclusion criteria that included “HIV-2 infection alone, pregnancy or not willing to practice barrier method of birth control” (pg. 3) [39]. Of the remaining four RCTs that excluded pregnant/breastfeeding women or women of child-bearing age, two RCTs were non-pharmacological interventions, one surgical, and one organizational intervention. No studies discuss the rationale or risk considerations for excluding on the basis of pregnancy.

#### Intervention description in RCTs

Only one mixed-sex RCT took sex or gender into account when describing the characteristics of how an intervention was implemented. In that study, authors reported using a “male and female team” to present educational sessions on managing side effects of androgen deprivation therapy (ADT) and the impact on couples [40]. The rationale provided was: “so that attendees would understand that the program was meant to serve equally the concerns of the male patient and the female partner (in heterosexual couples) (pg. 228)” [40].

#### Description of participants in RCTs

Almost all RCTs (98%) reported demographic characteristics by sex or gender by identifying number of male/female participants in text or as part of their demographic table describing baseline characteristics.

##### Single-sex studies

Six of the 12 single-sex RCTs used terms related to gender (men, women, boys) to report the population studied and the other six RCTs use the terms male and/or female.

##### Mixed-sex studies

Fifteen of 88 RCTs used the term gender to describe the population and 16 RCTs used the terms ‘men/women’ to report the population demographics. Eighty-six RCTs reported the population demographics according to sex or gender. As noted above, two RCTs did not provide any description of population by sex but instead referred to participants as “patients” (*n* = 1) [41] or “nurses/care aides” (*n* = 1) [42].

#### Statistical analysis of RCTs across sex or gender

##### Mixed-sex studies

Twenty of the 88 RCTs (23%) reported analyses related to sex or gender in the methods section, but none included a comprehensive sex and gender analysis across stages of the research process [6]. These statistical methods included randomization stratified by sex or gender (3 RCTs) [43–45], adjusting for sex or gender as a covariate (11 RCTs) [37, 46–55] and subgroup analysis across sex or gender (6 RCTs) [56–61].

The three trials which stratified randomization by sex or gender did not report subgroup analyses across sex or gender [43–45]. All 11 RCTs that described an intention to adjust for sex or gender as a variable, adjusted for or included sex or gender as a covariate in their analysis model.

For the six RCTs that reported results of subgroup analyses across sex or gender mentioned above, five RCTs pre-specified the intention to conduct subgroup analysis by sex or gender in the methods section. Only four studies provided sex-disaggregated data across intervention and comparator arms [51, 58, 60, 62]. For example, a study looking at effects of Korean red ginseng provides the mean and standard deviation of the outcome for each intervention arm and control arm disaggregated for male and female (pg. 166) [51]. Only one of the six studies that conducted subgroup analyses discussed the treatment interactions by sex in their discussion of results [60].

#### Discussion/conclusions of RCTs

##### Single-sex studies

Seven of 12 RCTs reported on sex or gender in their discussion regarding applicability and implications. For example, one study examining the effect of zinc supplementation on copper status in boys reported that “… the results of this study are expected to be generalizable to girls (pg. 288)” [63].

##### Mixed-sex studies

Eleven of the 88 RCTs reported on sex or gender implications of their findings. For example, one study stated that: “…although differences did not attain statistical significance, women were overrepresented in the SBI (ed: spiritually based intervention) and the extent to which findings can be fully extended to men is not clear (pg.504)” [64]. A study on colorectal cancer screening concluded: “Our results confirm those reported in that FOBT (ed: fecal occult blood test) uptake tends to increase with age among men and women (pg.9)” [65].

## Discussion

We found no examples of sex and gender analysis in a sample of 100 Canadian-led or funded RCTs [24, 27, 66]. Where sex and gender were considered, these considerations were often limited and mainly focused on biomedical analysis of differences across sex. For example, no studies considered the influence of gender. Only 6% (six studies) of our sample reported subgroup analyses across sex or gender. This was despite the fact that over 50% of our sample of RCTs evaluated non-pharmacologic interventions such as decision aids, cognitive therapy, self-help education tools, and community-based interventions where gender may play an important role in how the intervention is delivered (by whom and in what context) and received. Furthermore, of the six RCTs with subgroup analysis, only one commented in any depth on the methodological challenges of conducting sub-group analysis or on the significance of their findings and implications for clinical practice. We also noted inconsistent use of terminology in some RCTs, with sex and gender being used without definition and sometimes interchangeably. In RCTs, information on eligibility and recruitment forms is usually collected as “male/female”, and the use of the term “gender” may be inaccurate. These findings are consistent with those of recent studies looking at sex-related reporting in randomized trials published in major medical journals [23, 67]. Clarity in the application of the conceptual constructs of sex and gender and of sex and gender analysis is an important component of scientific rigor and contributes to the growing understanding of the ways that sex-based biological factors and gendered social factors are intertwined and interact with other social factors, shaping health behavior, opportunities, and outcomes [5, 24, 68–70].

The credibility and clinical importance of sex-based subgroup analyses must be carefully scrutinized since there is a risk of spurious findings due to under-powered tests and multiple testing. A recent meta-synthesis found that statistically significant effects of sex on treatment response in randomized trials were rarely reported, and when they were, they were rarely confirmed in subsequent trials or in meta-analyses, and very rarely led to a recommendation for differential treatment [71]. However, examples where differential treatment has been recommended (e.g., weight management for men by Public Health England [72], statin management [71], and differential dosage recommendations based on a differential risk-benefit ratio for Ambien (Zolpidem) [73], justify the need for sex and gender analysis in trials. The lack of data and analysis about women and coronary heart disease (due to exclusion of women from some clinical trials) is postulated as one of the reasons for under-treatment of women with symptoms of ischaemic heart disease [74]. There is a need for an improved evidence-base and for robust methodologies to determine differences and similarities of effects across sex and gender [6].

A variety of initiatives are attempting to address inconsistent terminology and the lack of robust sex and gender analysis in health research. For example, the Canadian Institutes of Health Research has published a casebook [66] and a tool for peer reviewers of grants and/or papers [27], a major advancement in promoting appropriate sex and gender analysis in research [75]. The SAGER (Sex and Gender Equity in Research) guidelines were developed by the Gender Policy Committee of the European Association of Science Editors (EASE) for reporting sex and gender in all types of science publications [31]. At the same time, academic journals are putting in place editorial policies that require sex-specific or gender-specific reporting [76]. Members of our team have recently developed reporting guidelines for health equity relevant RCTs, as an extension of the CONSORT statement (Consolidated Reporting of Randomized Trials) [77] and briefing notes to improve the consideration of sex and gender in systematic reviews [3]. All of these tools can be used by authors of RCTs to improve reporting of sex and gender.

More initiatives are needed to implement these and other tools and encourage their use to address the lack of sex and gender analysis in trials. Different strategies may be needed to tailor these initiatives for specific audiences such as trialists, funders, journal editors, patients/public, and policy-makers [78].

Furthermore, in our study, there were no RCTs that mentioned gender diverse populations. As knowledge of sex and gender analysis develops, approaches to study design and analysis need to keep current with clear and consistent terminology to support gender-sensitive decision-making [6].

This study examined reporting of sex, gender, and sex and gender analysis in a sample of Canadian RCTs, where sectors of the funding environment are actively promoting inclusion of sex and gender analysis in all research [26]. Although our study reviewed only a small cross-section of Canadian RCTs, our findings suggest that despite policies to encourage sex and gender analysis we are not yet seeing impacts in terms of sex and gender reporting in RCTs and uptake of sex and gender analysis. Reporting sex and gender considerations in peer-reviewed publications is a key indicator of whether policy efforts to mandate and encourage sex and gender analysis are effective. Thus, it will be important to continue to monitor changes in reporting over time to determine whether policy interventions are delivering their intended outcomes and develop new approaches for increasing skill level in the research and knowledge user communities.

This preliminary study has a number of limitations. This study is limited to what is reported in the included RCTs. It is possible that the effects of sex and/or gender on response were tested in more studies but not reported. Also, this sample of RCTs includes relatively small RCTs (with a median of 100 participants) and many of the studies would be underpowered to detect differences in response across sex or gender. In the six RCTs which did conduct a subgroup analysis across sex or gender, we did not assess the power for this analysis, nor the quality of these analyses because we expected there to be too few studies with these analyses to be generalizable to other situations. Another limitation is that our definition of “Canadian” trials may have missed or under-selected multi-national trials. We did not collect whether the flow of participants was reported according to gender (e.g., recruited, enrolled, completed), and this information would be important for conducting a sex and gender-based analysis. We also did not contact authors to obtain any additional details about sex and gender analysis, methods, or results. As well, the findings in this study may not be extrapolated to RCTs conducted primarily in other countries, due to the focus on Canadian RCTs, although some studies included multiple non-Canadian sites [23, 79].

## Conclusions

This survey of a sample of Canadian RCTs reveals very little analysis of differences or similarities in health outcomes across sex and/or gender, and no clear attempts on the part of researchers to integrate elements of sex and gender analysis. Furthermore, there was no mention of inclusion or exclusion of gender diverse people. This study provides a baseline and methodological approach to compare and assess changes in reporting about sex and gender and in the application of sex and gender analysis in future Canadian RCTs, and adds to global and Canadian efforts to make the case for integrating sex and gender analysis in health research. This study demonstrates a need for continued efforts to improve appropriate consideration and reporting of sex and gender and the integration of sex and gender analysis in randomized trials so that ultimately health services and policies address the needs of diverse populations and improve health outcomes for all.

## Additional files

Additional file 1: List of Data Extraction Items. (DOCX 19 kb)

Additional file 2: List of Funding Sources. (DOCX 21 kb)

Additional file 3: Table of Included Studies. (DOCX 49 kb)

## Abbreviations

CIHR: Canadian Institutes of Health Research
EASE: European Association of Science Editors
NIH: National Institute of Health
PRISMA-Equity: Preferred Reporting Items for Systematic Reviews and Meta-Analyses for equity-focused systematic reviews
RCT: Randomized controlled trials
