# Supplementary material for: Reporting of sex and gender in randomized controlled trials in Canada: a cross-sectional methods study
Source: Res Integr Peer Rev. 2017 Sep 1;2:15. doi: 10.1186/s41073-017-0039-6 (PMC5803639; doi:10.1186/s41073-017-0039-6)
Supplement: Supplementary file 1 — List of Data Extraction Items. (DOCX 19 kb) [file 41073_2017_39_MOESM1_ESM.docx]

**Appendix 1: List of Data Extraction Items**

1. Author name and year
2. Title of Study
3. RCT (yes/No)
4. Data extractor (initials)
5. PDF available
6. Population (how was population defined?)
7. Is population defined as disadvantaged or vulnerable across PROGRESS+?
8. Intervention (what was the intervention?)
9. Type of Intervention
10. Is intervention: 1) targeted to disadvantaged population; 2) universal (aimed at everyone); 3) aimed at reducing health inequities across a social gradient?
11. Comparison (is it compared to another intervention or usual care or placebo?)
12. Study design: cluster RCT or individual RCT, If cluster RCT, what was the unit of allocation? (e.g. schools, health clinics)
13. Study design: Cross-over or parallel group RCT
14. Describe the site: multi-site or single site; within Canada or outside of Canada,
15. Primary outcome: does RCT describe primary outcome?
16. Primary outcome: list primary outcome if specified.
17. If not, list all other outcomes, Adverse effects or safety outcomes
18. Title/Abstract: is sex/gender mentioned in the title or abstract? (Y/N)
19. Title/Abstract: Copy text about sex/gender
20. Introduction: was sex/gender discussed in the introduction as being relevant to effectiveness or outcomes?
21. Introduction: copy text
22. Methods: did the authors report how sex/gender was considered?
23. Methods: copy text,
24. Eligibility criteria: are any people excluded on the basis of sex/gender?
25. Eligibility criteria: describe eligibility criteria related to sex/gender (if any)
26. Recruitment methods, are they described?
27. Recruitment methods: paste description
28. Ethical consent procedure described?
29. Ethical consent procedure: how was ethics described?
30. Sample size, total number randomized
31. Sample size, total number analyzed
32. Population: was population reported according to sex/gender (Y/N)?
33. Number of population male: provide number of men
34. Number of population female: provide number of women
35. Population: how was population described by sex/gender? (text, and include the characteristics- e.g. 50% female or Male: female ratio: 0.8) copy text on gender description (if not available write "nr or tabulated form)
36. Characteristics of population: Place of residence (urban, rural, inner city, other?)
37. Characteristics of population: Race/ethnicity/ culture/ language
38. Characteristics of population: Occupation
39. Characteristics of population: Religion
40. Characteristics of population: Education
41. Characteristics of population: Socioeconomic status
42. Characteristics of population: Social capital
43. Characteristics of population: Plus of PROGRESS: any details about other personal characteristics associated with inequity such as age, disability)
44. Characteristics of population: Plus of PROGRESS 2: any details about relationships affecting inequity (e.g. historical context, family environment, school setting)
45. Characteristics of population: Plus of PROGRESS 3: any details about time-dependent settings that may affect inequity, e.g. discharge from hospital or jail?
46. Results: was data disaggregated by sex/gender?
47. Results: copy text about sex/gender analysis
48. Subgroup analysis justification: was there a justification (e.g. based on a theory or previous finding) for subgroup analysis?
49. Subgroup analysis justification: copy text, was a subgroup analysis or a meta regression or any sort of modeling approach carried out based on sex/gender?
50. Subgroup analysis: copy text
51. Subgroup analysis: Place of residence (urban, rural, inner city, other?)
52. Subgroup analysis: Race/ethnicity/ culture/ language
53. Subgroup analysis: Occupation
54. Subgroup analysis: Religion
55. Subgroup analysis: Education
56. Subgroup analysis: Socioeconomic status
57. Subgroup analysis: Social capital
58. Subgroup analysis: Plus of PROGRESS: any subgroup analyses across personal characteristics associated with inequity such as age, disability)
59. Subgroup analysis: Plus of PROGRESS 2: any details about analysis regarding relationships affecting inequity (e.g. historical context, family environment, school setting)
60. Subgroup analysis: Plus of PROGRESS 3: any details about analysis regarding time-dependent settings that may affect inequity, e.g. discharge from hospital or jail?
61. Applicability or generalisability discussed with respect to sex/gender?
62. Applicability: how was applicability discussed regarding sex/gender?
63. EQUITY: how was inequity assessed across the PROGRESS factors (place of residence, race, occupation, gender, religion, educational achievement, socio economic status or social capital) EQUITY: how were political, historical, cultural and/or social issues factored into the analysis of equity? Theory: what theory related to sex/gender (usually in the study design or abstract) formed the author's orientation for the study? Indicate 'not reported' if no theory was considered. Canadian first institution (y/n)
64. Canadian last institution (y/n)
65. Canadian funding (y/n)
66. Canadian funder (sole funder or one of the multiple funders Multiple funders?
67. Industry/Government/Nonprofit
