# Supplementary material for: Reporting of sex and gender in randomized controlled trials in Canada: a cross-sectional methods study
Source: Res Integr Peer Rev. 2017 Sep 1;2:15. doi: 10.1186/s41073-017-0039-6 (PMC5803639; doi:10.1186/s41073-017-0039-6)
Supplement: Supplementary file 2 — List of Funding Sources. (DOCX 21 kb) [file 41073_2017_39_MOESM2_ESM.docx]

**Appendix 2: List of Funding Sources**

| **Name of Funding Source** | **Number of Trials Funded** |
| --- | --- |
| Alberta Innovates Health Solutions studentship | 1 |
| American Shoulder and Elbow Surgeons Grant. | 1 |
| Boeringher Inglheim | 3 |
| Canadian Institute of Health Research | 21 |
| In-kind support from Copan Italia, Brescia Italy. | 1 |
| Southwest Oncology Group Grant | 1 |
| The International MDT Research Foundation | 1 |
| Abbott Laboratories, Canada | 1 |
| Academic Physician's Fund | 1 |
| Actelion Pharmaceuticals | 2 |
| Adiga Life Sciences and Circassia (UK) | 1 |
| AETMIS-CHU Sainte-Justine Research Centre, the Canadian Pain Society-Purdue Pharma (Interprofessional Research Award). | 1 |
| Agence de la Santé et des Services Sociaux de l’Abitibi-Témiscamingue | 1 |
| Agriculture and Agri-Food Canada | 1 |
| Alberta Cancer Foundation | 1 |
| AstraZeneca | 2 |
| BC Centre for Disease Control | 1 |
| British Colombia Lung Association | 1 |
| British Columbia Medical Service | 1 |
| Canada Foundation for Innovation | 1 |
| Canada manufactures of Telmisartan | 1 |
| Canada Research Chairs | 1 |
| Canadian Anesthesia Research Foundation. | 1 |
| Canadian Arthritis Network | 1 |
| Canadian Breast Cancer foundation | 1 |
| Canadian Breast Cancer Research Alliance | 1 |
| Canadian Cancer Society, Alberta Division | 1 |
| Canadian Health Services Research Foundation | 1 |
| Canadian Network and Centre for Trials Internationally (CANNeCTIN) | 1 |
| Canadian Stroke Network | 1 |
| Canola Council of Canada | 1 |
| CIBC World Markets Children’s Miracle Foundation Endowed Chair in Child Health Research | 1 |
| Community Clinical Oncology Program Grant | 1 |
| Dalhousie University, Department of Anaesthesia | 1 |
| Deanship of Scientific Research at King Saud University: Saudi Arabia | 1 |
| Dr Tony Hakim Innovative Stroke Research Award | 1 |
| Eastern Cooperative Oncology Group Grants | 1 |
| Government of Quebec | 1 |
| Groupe de Recherche Interuniversitaire en Interventions en Sciences Infirmieres du Quebec (GRIISIQ) | 1 |
| Hamilton Academic Health Sciences Organization (HAHSO) alternative Funding Plan (AFP) Innovation Fund. | 1 |
| Health Canada | 2 |
| Heart and Stroke Canada | 1 |
| Heart and Stroke Foundation Centre for Stroke Recovery | 1 |
| Heart and Stroke Foundation of Ontario | 5 |
| Heart and Stroke Foundation of Quebec | 1 |
| Hopewell Professorship | 1 |
| Loblaw Companies | 1 |
| Manitoba Medical Service Association | 1 |
| Medtronic | 2 |
| Mental Health Commission of Canada. | 1 |
| Ministries of Health of British Columbia | 1 |
| Ministries of Health of Nova Scotia | 1 |
| Ministries of Health of Quebec | 1 |
| National Cancer Institute | 1 |
| National Heart Foundation of Australia project grant | 1 |
| North Central Cancer Treatment Group Grant | 1 |
| Nova Scotia Health Research Foundation | 1 |
| Ontario HIV Treatment Network (OHTN) | 1 |
| Ontario Institute for Cancer Research | 1 |
| Ontario Ministry of Health Promotion | 1 |
| Ontario Neurotrauma Foundation | 1 |
| Ottawa Hospital Women’s Breast Health Centre | 1 |
| Pacific Alzheimer’s Research Foundation | 1 |
| Personal donation from Tony and Elizabeth Graham. | 1 |
| Phase III Academic Health Science Centre/Alternate Funding Plan Innovation Fund | 1 |
| Physicians Services Incorporated Foundation | 1 |
| Providence Health Care | 1 |
| Public Health Agency of Canada | 1 |
| Radiation Therapy Oncology Group Grants | 1 |
| Resident Research Support Grant | 1 |
| Sanofi-Aventis | 1 |
| Saskatchewan Health Research Foundation | 1 |
| Schizophrenia Society of Ontario | 1 |
| Social Sciences and Humanities Research Council | 1 |
| St Michaels Hospital | 1 |
| St. Jude Canada. | 1 |
| Sunnybrook Health Sciences Centre, Department of Anesthesia | 1 |
| The 2010 Canadian Anesthesiologists’ Society/ GE Healthcare Award | 1 |
| The Drummond Foundation and the Ontario Research Coalition. | 1 |
| The Henderson Research Centre, Hamilton | 1 |
| The National Institutes of Health. | 1 |
| Toronto General and Western Hospital Foundation, Toronto, Canada | 2 |
| Universite de Montreal | 1 |
| University of British Columbia | 1 |
| University of Calgary | 2 |
| University of Manitoba Health Sciences Center Research Foundation and Department of Research | 1 |
| University of Melbourne (MAH), Early Career Researcher Project Grant | 1 |
| University of Ottawa | 4 |
| University of Toronto | 5 |
| Unrestricted Covidien grant | 1 |
| Vancouver Coastal Authority | 1 |
| Not Reported | 19 |
