# Supplementary material for: Reporting of sex and gender in randomized controlled trials in Canada: a cross-sectional methods study
Source: Res Integr Peer Rev. 2017 Sep 1;2:15. doi: 10.1186/s41073-017-0039-6 (PMC5803639; doi:10.1186/s41073-017-0039-6)
Supplement: Supplementary file 3 — Table of Included Studies. (DOCX 49 kb) [file 41073_2017_39_MOESM3_ESM.docx]

**Appendix 3: Table of Included Studies**

| 1. Aaron SD, Vandemheen KL, Maltais F, Field SK, Sin DD, Bourbeau J, Marciniuk DD, FitzGerald JM, Nair P, Mallick R: TNFalpha antagonists for acute exacerbations of COPD: a randomised double-blind controlled trial. Thorax 2013, 68(2):142-148. |
| --- |
| 1. Anandakrishnan S, Balki M, Farine D, Seaward G, Carvalho JC: Carbetocin at elective Cesarean delivery: a randomized controlled trial to determine the effective dose, part 2. Canadian Journal of Anaesthesia, 2013, 60(11):1054-1060. |
| 1. Andersen LL, Jay K, Andersen CH, Jakobsen MD, Sundstrup E, Topp R, Behm DG: Acute effects of massage or active exercise in relieving muscle soreness: randomized controlled trial. Journal of Strength and Conditioning Research 2013, 27(12):3352-3359. |
| 1. Armstrong PW, Gershlick AH, Goldstein P, Wilcox R, Danays T, Lambert Y, Sulimov V, Rosell Ortiz F, Ostojic M, Welsh RC et al: Fibrinolysis or primary PCI in ST-segment elevation myocardial infarction. The New England Journal of Medicine 2013, 368(15):1379-1387. |
| 1. Aronson R, Cohen O, Conget I, Runzis S, Castaneda J, de Portu S, Lee S, Reznik Y: OpT2mise: a randomized controlled trial to compare insulin pump therapy with multiple daily injections in the treatment of type 2 diabetes-research design and methods. Diabetes Technology & Therapeutics 2014, 16(7):414-420. |
| 1. Atzema CL, Austin PC, Wu L, Brzozowski M, Feldman MJ, McDonnell M, Mazurik L: Speak fast, use jargon, and don't repeat yourself: a randomized trial assessing the effectiveness of online videos to supplement emergency department discharge instructions. PloS One 2013, 8(11):e77057. |
| 1. Awad IT, Cheung JJ, Al-Allaq Y, Conroy PH, McCartney CJ: Low-dose spinal bupivacaine for total knee arthroplasty facilitates recovery room discharge: a randomized controlled trial. Canadian Journal of Anaesthesia, 2013, 60(3):259-265. |
| 1. Balfour L, Spaans JN, Fergusson D, Huff H, Mills EJ, la Porte CJ, Walmsley S, Singhal N, Rosenes R, Tremblay N et al: Micronutrient Deficiency and Treatment Adherence in a Randomized Controlled Trial of Micronutrient Supplementation in ART-Naïve Persons with HIV. PloS One 2014, 9(1):e85607. |
| 1. Barkun AN, Bhat M, Armstrong D, Dawes M, Donner A, Enns R, Martin J, Moayyedi P, Romagnuolo J, Stitt L: Effectiveness of disseminating consensus management recommendations for ulcer bleeding: a cluster randomized trial. CMAJ : Canadian Medical Association Journal, 2013, 185(3):E156-166. |
| 1. Bedard M, Felteau M, Marshall S, Cullen N, Gibbons C, Dubois S, Maxwell H, Mazmanian D, Weaver B, Rees L et al: Mindfulness-based cognitive therapy reduces symptoms of depression in people with a traumatic brain injury: results from a randomized controlled trial. The Journal of Head Trauma Rehabilitation 2014, 29(4):E13-22. |
| 1. Bertinato J, Simpson JR, Sherrard L, Taylor J, Plouffe LJ, Van Dyke D, Geleynse M, Dam YY, Murphy P, Knee C et al: Zinc supplementation does not alter sensitive biomarkers of copper status in healthy boys. The Journal of Nutrition 2013, 143(3):284-289. |
| 1. Bissonnette R, Tardif JC, Harel F, Pressacco J, Bolduc C, Guertin MC: Effects of the tumor necrosis factor-alpha antagonist adalimumab on arterial inflammation assessed by positron emission tomography in patients with psoriasis: results of a randomized controlled trial. Circulation Cardiovascular Imaging 2013, 6(1):83-90. |
| 1. Boivin A, Lehoux P, Lacombe R, Burgers J, Grol R: Involving patients in setting priorities for healthcare improvement: a cluster randomized trial. Implementation Science 2014, 9(1):24. |
| 1. Brahmania M, Ou G, Bressler B, Ko HK, Lam E, Telford J, Enns R: 2 L versus 4 L of PEG3350 + electrolytes for outpatient colonic preparation: a randomized, controlled trial. Gastrointestinal Endoscopy 2014, 79(3):408-416 e404. |
| 1. Cairncross G, Wang M, Shaw E, Jenkins R, Brachman D, Buckner J, Fink K, Souhami L, Laperriere N, Curran W et al: Phase III trial of chemoradiotherapy for anaplastic oligodendroglioma: long-term results of RTOG 9402. Journal of Clinical Oncology, 2013, 31(3):337-343. |
| 1. Campbell-Yeo ML, Johnston CC, Joseph KS, Feeley N, Chambers CT, Barrington KJ, Walker CD: Co-bedding between preterm twins attenuates stress response after heel lance: results of a randomized trial. The Clinical Journal of Pain 2014, 30(7):598-604. |
| 1. Carlson LE, Waller A, Groff SL, Bultz BD: Screening for distress, the sixth vital sign, in lung cancer patients: effects on pain, fatigue, and common problems--secondary outcomes of a randomized controlled trial. Psycho-oncology 2013, 22(8):1880-1888. |
| 1. Carlsten C, Dimich-Ward H, Ferguson A, Watson W, Rousseau R, Dybuncio A, Becker A, Chan-Yeung M: Atopic dermatitis in a high-risk cohort: natural history, associated allergic outcomes, and risk factors. Annals of Allergy, Asthma & Immunology, 2013, 110(1):24-28.   ** Chan-Yeung M, Manfreda J, Dimich-Ward H, Ferguson A, Watson W, Becker A: A randomized controlled study on the effectiveness of a multifaceted intervention program in the primary prevention of asthma in high-risk infants. Archives of Pediatrics & Adolescent Medicine 2000, 154(7):657-663. |
| 1. Celebrini RG, Eng JJ, Miller WC, Ekegren CL, Johnston JD, Depew TA, Macintyre DL: Effect of a novel movement strategy in decreasing ACL risk factors in female adolescent soccer players: a randomized controlled trial. Clinical Journal of Sport Medicine 2014, 24(2):134-141. |
| 1. Chandler JR, Myers D, Mehta D, Whyte E, Groberman MK, Montgomery CJ, Ansermino JM: Emergence delirium in children: a randomized trial to compare total intravenous anesthesia with propofol and remifentanil to inhalational sevoflurane anesthesia. Paediatric Anaesthesia 2013, 23(4):309-315. |
| 1. Chetty VT, Damjanovic S, Gerstein H, Singh N, Yusuf S, Anand SS, Sharma AM: Metabolic effects of telmisartan in subjects with abdominal obesity: a prospective randomized controlled trial. Blood Pressure 2014, 23(1):54-60. |
| 1. Chiu M, Bryson GL, Lui A, Watters JM, Taljaard M, Nathan HJ: Reducing persistent postoperative pain and disability 1 year after breast cancer surgery: a randomized, controlled trial comparing thoracic paravertebral block to local anesthetic infiltration. Annals of Surgical Ooncology 2014, 21(3):795-801. |
| 1. Choi S, Rampersaud YR, Chan VW, Persaud O, Koshkin A, Tumber P, Brull R: The addition of epidural local anesthetic to systemic multimodal analgesia following lumbar spinal fusion: a randomized controlled trial. Canadian Journal of Anaesthesia, 2014, 61(4):330-339. |
| 1. Clouston K, Katz A, Martens PJ, Sisler J, Turner D, Lobchuk M, McClement S: Does access to a colorectal cancer screening website and/or a nurse-managed telephone help line provided to patients by their family physician increase fecal occult blood test uptake?: A pragmatic cluster randomized controlled trial study protocol. BMC Cancer 2012, 12:182. |
| 1. Coyle D, Grunfeld E, Coyle K, Pond G, Julian JA, Levine MN: Cost effectiveness of a survivorship care plan for breast cancer survivors. Journal of Oncology Practice 2014, 10(2):e86-e92.   **Grunfeld E, Julian JA, Pond G, Maunsell E, Coyle D, Folkes A, Joy AA, Provencher L, Rayson D, Rheaume DE et al: Evaluating survivorship care plans: results of a randomized, clinical trial of patients with breast cancer. Journal of Clinical Oncology 2011, 29(36):4755-4762. |
| 1. Davis EM, Lynd LD, Grubisic M, Kopec JA, Sayre EC, Cibere J, Esdaile J, Marra CA: Responsiveness of health state utility values in knee osteoarthritis. The Journal of Rheumatology 2013, 40(12):2075-2082.   ** Marra CA, Cibere J, Grubisic M, Grindrod KA, Gastonguay L, Thomas JM, Embley P, Colley L, Tsuyuki RT, Khan KM et al: Pharmacist-initiated intervention trial in osteoarthritis: a multidisciplinary intervention for knee osteoarthritis. Arthritis Care & Research 2012, 64(12):1837-1845. |
| 1. Dawson D, Richardson J, Troyer A, Binns M, Clark A, Polatajko H, Winocur G, Hunt A, Bar Y: An occupation-based strategy training approach to managing age-related executive changes: a pilot randomized controlled trial. Clinical Rehabilitation 2014, 28(2):118-127. |
| 1. Day V, McGrath PJ, Wojtowicz M: Internet-based guided self-help for university students with anxiety, depression and stress: a randomized controlled clinical trial. Behaviour Research and Therapy 2013, 51(7):344-351. |
| 1. Dixon A, Clarkin C, Barrowman N, Correll R, Osmond MH, Plint AC: Reduction of radial-head subluxation in children by triage nurses in the emergency department: a cluster-randomized controlled trial. CMAJ : Canadian Medical Association Journal 2014, 186(9):E317-E323. |
| 1. Dobson SR, McNeil S, Dionne M, Dawar M, Ogilvie G, Krajden M, Sauvageau C, Scheifele DW, Kollmann TR, Halperin SA et al: Immunogenicity of 2 doses of HPV vaccine in younger adolescents vs 3 doses in young women: a randomized clinical trial. Jama 2013, 309(17):1793-1802. |
| 1. Eisenberg MJ, Grandi SM, Gervais A, O'Loughlin J, Paradis G, Rinfret S, Sarrafzadegan N, Sharma S, Lauzon C, Yadav R et al: Bupropion for smoking cessation in patients hospitalized with acute myocardial infarction: a randomized, placebo-controlled trial. Journal of the American College of Cardiology 2013, 61(5):524-532. |
| 1. Ferguson ND, Cook DJ, Guyatt GH, Mehta S, Hand L, Austin P, Zhou Q, Matte A, Walter SD, Lamontagne F et al: High-frequency oscillation in early acute respiratory distress syndrome. The New England Journal of Medicine 2013, 368(9):795-805. |
| 1. Fiest KM, Sajobi TT, Wiebe S: Epilepsy surgery and meaningful improvements in quality of life: results from a randomized controlled trial. Epilepsia 2014, 55(6):886-892. |
| 1. Flook NW, Moayyedi P, Dent J, Talley NJ, Persson T, Karlson BW, Ruth M: Acid-suppressive therapy with esomeprazole for relief of unexplained chest pain in primary care: a randomized, double-blind, placebo-controlled trial. The American Journal of Gastroenterology 2013, 108(1):56-64. |
| 1. Gan EC, Habib AR, Rajwani A, Javer AR: Five-degree, 10-degree, and 20-degree reverse Trendelenburg position during functional endoscopic sinus surgery: a double-blind randomized controlled trial. International Forum of Allergy & Rhinology 2014, 4(1):61-68. |
| 1. Ghandehari OO, Hadjistavropoulos T, Williams J, Thorpe L, Alfano DP, Dal Bello-Haas V, Malloy DC, Martin RR, Rahaman O, Zwakhalen SM et al: A controlled investigation of continuing pain education for long-term care staff. Pain Research & Management 2013, 18(1):11-18. |
| 1. Gillingham LG, Harding SV, Rideout TC, Yurkova N, Cunnane SC, Eck PK, Jones PJ: Dietary oils and FADS1-FADS2 genetic variants modulate [13C]alpha-linolenic acid metabolism and plasma fatty acid composition. The American Journal of Clinical Nutrition 2013, 97(1):195-207. |
| 1. Glicksman JT, Sherman I, Rotenberg BW: Informed consent when prescribing medication: a randomized controlled trial. The Laryngoscope 2014, 124(6):1296-1300. |
| 1. Goeree R, von Keyserlingk C, Burke N, He J, Kaczorowski J, Chambers L, Dolovich L, Michael Paterson J, Zagorski B: Economic appraisal of a community-wide cardiovascular health awareness program. Value in health : the journal of the International Society for Pharmacoeconomics and Outcomes Research 2013, 16(1):39-45.   ** Kaczorowski J, Chambers LW, Dolovich L, et al. Improving cardiovascular health at population level: 39 community cluster randomised trial of Cardiovascular Health Awareness Program (CHAP). The BMJ. 2011;342:d442. doi:10.1136/bmj.d442. |
| 1. Goodall EC, Granados AC, Luinstra K, Pullenayegum E, Coleman BL, Loeb M, Smieja M: Vitamin D3 and gargling for the prevention of upper respiratory tract infections: a randomized controlled trial. BMC Infectious Diseases 2014, 14:273. |
| 1. Hartling L, Scott SD, Johnson DW, Bishop T, Klassen TP: A randomized controlled trial of storytelling as a communication tool. PloS One 2013, 8(10):e77800. |
| 1. Hassanain M, Metrakos P, Fisette A, Doi SA, Schricker T, Lattermann R, Carvalho G, Wykes L, Molla H, Cianflone K: Randomized clinical trial of the impact of insulin therapy on liver function in patients undergoing major liver resection. The British Journal of Surgery 2013, 100(5):610-618.   ** Sato H, Lattermann R, Carvalho G, Sato T, Metrakos P, Hassanain M et al. Perioperative glucose and insulin administration while maintaining normoglycemia (GIN therapy) in patients undergoing major liver resection. Anesthesia and analgesia. 2010;110(6):1711-8. |
| 1. Hathorn IF, Habib AR, Manji J, Javer AR: Comparing the reverse Trendelenburg and horizontal position for endoscopic sinus surgery: a randomized controlled trial. Otolaryngology--head and neck surgery : official journal of American Academy of Otolaryngology-Head and Neck Surgery 2013, 148(2):308-313. |
| 1. Hayter M, Bould M, Afsari M, Riem N, Chiu M, Boet S: Does warm-up using mental practice improve crisis resource management performance? A simulation study. British Journal of Anaesthesia 2013, 110(2):299-304. |
| 1. Hogan ME, Probst J, Wong K, Riddell RP, Katz J, Taddio A: A randomized-controlled trial of parent-led tactile stimulation to reduce pain during infant immunization injections. The Clinical Journal of Pain 2014, 30(3):259-265. |
| 1. Holmqvist M, Vincent N, Walsh K: Web- vs. telehealth-based delivery of cognitive behavioral therapy for insomnia: a randomized controlled trial. Sleep Medicine 2014, 15(2):187-195. |
| 1. Hopman WM, VanDenKerkhof EG, Carley ME, Kuhnke JL, Harrison MB: Factors associated with health-related quality of life in chronic leg ulceration. Quality of Life Research : an International Journal of Quality of Life Aspects of Treatment, Care and Rehabilitation 2014, 23(6):1833-1840.   ** Harrison MB, Vandenkerkhof EG, Hopman WM, Graham ID, Carley ME, Nelson EA: The Canadian Bandaging Trial: Evidence-informed leg ulcer care and the effectiveness of two compression technologies. BMC Nursing 2011, 10:20. |
| 1. Hoppe DJ, Denkers M, Hoppe FM, Wong IH: The use of video before arthroscopic shoulder surgery to enhance patient recall and satisfaction: a randomized-controlled study. Journal of Shoulder and Elbow Surgery 2014, 23(6):e134-139. |
| 1. Hunt MA, Keefe FJ, Bryant C, Metcalf BR, Ahamed Y, Nicholas MK, Bennell KL: A physiotherapist-delivered, combined exercise and pain coping skills training intervention for individuals with knee osteoarthritis: a pilot study. The Knee 2013, 20(2):106-112. |
| 1. Hurlbert RJ, Alexander D, Bailey S, Mahood J, Abraham E, McBroom R, Jodoin A, Fisher C: rhBMP-2 for posterolateral instrumented lumbar fusion: a multicenter prospective randomized controlled trial. Spine 2013, 38(25):2139-2148. |
| 1. Janssen PA, Weissinger S: Women's perception of pre-hospital labour duration and obstetrical outcomes; a prospective cohort study. BMC Pregnancy and Childbirth 2014, 14:182.   **Janssen PA, Still DK, Klein MC, Singer J, Carty EA, Liston RM, Zupancic JA: Early labor assessment and support at home versus telephone triage: a randomized controlled trial. Obstetrics and Gynecology 2006, 108(6):1463-1469. |
| 1. Jenkins DJ, Kendall CW, Vuksan V, Faulkner D, Augustin LS, Mitchell S, Ireland C, Srichaikul K, Mirrahimi A, Chiavaroli L et al: Effect of lowering the glycemic load with canola oil on glycemic control and cardiovascular risk factors: a randomized controlled trial. Diabetes Care 2014, 37(7):1806-1814. |
| 1. Jovanovski E, Peeva V, Sievenpiper JL, Jenkins AL, Desouza L, Rahelic D, Sung MK, Vuksan V: Modulation of endothelial function by Korean red ginseng (Panax ginseng C.A. Meyer) and its components in healthy individuals: a randomized controlled trial. Cardiovascular Therapeutics 2014, 32(4):163-169. |
| 1. Kidd SA, Kaur J, Virdee G, George TP, McKenzie K, Herman Y: Cognitive remediation for individuals with psychosis in a supported education setting: a randomized controlled trial. Schizophrenia Research 2014, 157(1-3):90-98. |
| 1. Koszycki D, Bilodeau C, Raab-Mayo K, Bradwejn J: A multifaith spiritually based intervention versus supportive therapy for generalized anxiety disorder: a pilot randomized controlled trial. Journal of Clinical Psychology 2014, 70(6):489-509. |
| 1. Kurashima Y, Feldman LS, Kaneva PA, Fried GM, Bergman S, Demyttenaere SV, Li C, Vassiliou MC: Simulation-based training improves the operative performance of totally extraperitoneal (TEP) laparoscopic inguinal hernia repair: a prospective randomized controlled trial. Surgical Endoscopy 2014, 28(3):783-788. |
| 1. Lamy A, Devereaux PJ, Prabhakaran D, Taggart DP, Hu S, Paolasso E, Straka Z, Piegas LS, Akar AR, Jain AR et al: Effects of off-pump and on-pump coronary-artery bypass grafting at 1 year. The New England Journal of Medicine 2013, 368(13):1179-1188. |
| 1. Le May S, Gouin S, Fortin C, Messier A, Robert MA, Julien M: Efficacy of an ibuprofen/codeine combination for pain management in children presenting to the emergency department with a limb injury: a pilot study. The Journal of Emergency Medicine 2013, 44(2):536-542. |
| 1. Leipsic J, LaBounty TM, Ajlan AM, Earls JP, Strovski E, Madden M, Wood DA, Hague CJ, Poulter R, Branch K et al: A prospective randomized trial comparing image quality, study interpretability, and radiation dose of narrow acquisition window with widened acquisition window protocols in prospectively ECG-triggered coronary computed tomography angiography. Journal of Cardiovascular Computed Tomography 2013, 7(1):18-24. |
| 1. Lewis NC, Ainslie PN, Atkinson G, Jones H, Grant EJ, Lucas SJ: Initial orthostatic hypotension and cerebral blood flow regulation: effect of alpha1-adrenoreceptor activity. American journal of physiology Regulatory, Integrative and Comparative Physiology 2013, 304(2):R147-154. |
| 1. Linkins LA, Bates SM, Lang E, Kahn SR, Douketis JD, Julian J, Parpia S, Gross P, Weitz JI, Spencer FA et al: Selective D-dimer testing for diagnosis of a first suspected episode of deep venous thrombosis: a randomized trial. Annals of Internal Medicine 2013, 158(2):93-100. |
| 1. Macdonald RL, Higashida RT, Keller E, Mayer SA, Molyneux A, Raabe A, Vajkoczy P, Wanke I, Bach D, Frey A et al: Randomised trial of clazosentan, an endothelin receptor antagonist, in patients with aneurysmal subarachnoid hemorrhage undergoing surgical clipping (CONSCIOUS-2). Acta neurochirurgica Supplement 2013, 115:27-31.   ** Macdonald RL, Higashida RT, Keller E, Mayer SA, Molyneux A, Raabe A et al. Clazosentan, an endothelin receptor antagonist, in patients with aneurysmal subarachnoid haemorrhage undergoing surgical clipping: a randomised, double-blind, placebo-controlled phase 3 trial (CONSCIOUS-2). The Lancet Neurology. 2011;10(7):618-25. |
| 1. Maksymowych WP, Morency N, Conner-Spady B, Lambert RG: Suppression of inflammation and effects on new bone formation in ankylosing spondylitis: evidence for a window of opportunity in disease modification. Ann Rheum Dis 2013, 72(1):23-28.   ** Lambert RG, Salonen D, Rahman P, Inman RD, Wong RL, Einstein SG, Thomson GT, Beaulieu A, Choquette D, Maksymowych WP: Adalimumab significantly reduces both spinal and sacroiliac joint inflammation in patients with ankylosing spondylitis: a multicenter, randomized, double-blind, placebo-controlled study. Arthritis and Rheumatism 2007, 56(12):4005-4014. |
| 1. Maltepe C, Koren G: Preemptive treatment of nausea and vomiting of pregnancy: results of a randomized controlled trial. Obstetrics and Gynecology International 2013, 2013:809787. |
| 1. Masson PC, von Ranson KM, Wallace LM, Safer DL: A randomized wait-list controlled pilot study of dialectical behaviour therapy guided self-help for binge eating disorder. Behaviour Research and Therapy 2013, 51(11):723-728. |
| 1. Mayich DJ, Tieszer C, Lawendy A, McCormick W, Sanders D: Role of patient information handouts following operative treatment of ankle fractures: a prospective randomized study. Foot & Ankle International 2013, 34(1):2-7. |
| 1. McCrindle BW, Manlhiot C, Cochrane A, Roberts R, Hughes M, Szechtman B, Weintraub R, Andrew M, Monagle P: Factors associated with thrombotic complications after the Fontan procedure: a secondary analysis of a multicenter, randomized trial of primary thromboprophylaxis for 2 years after the Fontan procedure. Journal of the American College of Cardiology 2013, 61(3):346-353.   **Monagle P, Cochrane A, Roberts R, Manlhiot C, Weintraub R, Szechtman B, Hughes M, Andrew M, McCrindle BW: A multicenter, randomized trial comparing heparin/warfarin and acetylsalicylic acid as primary thromboprophylaxis for 2 years after the Fontan procedure in children. Journal of the American College of Cardiology 2011, 58(6):645-651. |
| 1. McEwen D, Taillon-Hobson A, Bilodeau M, Sveistrup H, Finestone H: Virtual reality exercise improves mobility after stroke: an inpatient randomized controlled trial. Stroke; a Journal of Cerebral Circulation 2014, 45(6):1853-1855. |
| 1. McGowan CL, Murai H, Millar PJ, Notarius CF, Morris BL, Floras JS: Simvastatin reduces sympathetic outflow and augments endothelium-independent dilation in non-hyperlipidaemic primary hypertension. Heart (British Cardiac Society) 2013, 99(4):240-246. |
| 1. Mogollon JA, Boivin C, Lemieux S, Blanchet C, Claveau J, Dodin S: Chocolate flavanols and skin photoprotection: a parallel, double-blind, randomized clinical trial. Nutrition Journal 2014, 13:66. |
| 1. Nagamatsu LS, Chan A, Davis JC, Beattie BL, Graf P, Voss MW, Sharma D, Liu-Ambrose T: Physical activity improves verbal and spatial memory in older adults with probable mild cognitive impairment: a 6-month randomized controlled trial. Journal of Aging Research 2013, 2013:861893.   **Nagamatsu LS, Handy TC, Hsu CL, Voss M, Liu-Ambrose T: Resistance training promotes cognitive and functional brain plasticity in seniors with probable mild cognitive impairment: A 6-month randomized controlled trial. Archives of Internal Medicine 2012, 172(8):666-668. |
| 1. Nieuwlaat R, Eikelboom JW, Schulman S, van Spall HG, Schulze KM, Connolly BJ, Cuddy SM, Hubers LM, Stehouwer AC, Connolly SJ: Cluster randomized controlled trial of a simple warfarin maintenance dosing algorithm versus usual care among primary care practices. Journal of Thrombosis and Thrombolysis 2014, 37(4):435-442. |
| 1. Osana HP, Pitsolantis N: Addressing the struggle to link form and understanding in fractions instruction. The British Journal of Educational Psychology 2013, 83(Pt 1):29-56. |
| 1. Ou G, Kim E, Lakzadeh P, Tong J, Enns R, Ramji A, Whittaker S, Ko HH, Bressler B, Halparin L et al: A randomized controlled trial assessing the effect of prescribed patient position changes during colonoscope withdrawal on adenoma detection. Gastrointestinal Endoscopy 2014, 80(2):277-283. |
| 1. Ou G, Svarta S, Chan C, Galorport C, Qian H, Enns R: The effect of chewing gum on small-bowel transit time in capsule endoscopy: a prospective, randomized trial. Gastrointestinal Endoscopy, 79(4):630-636. |
| 1. Oviedo-Joekes E, Guh D, Marchand K, Marsh DC, Lock K, Brissette S, Anis AH, Schechter MT: Differential long-term outcomes for voluntary and involuntary transition from injection to oral opioid maintenance treatment. Substance Abuse Treatment, Prevention, and Policy 2014, 9(1):1.   **Oviedo-Joekes E, Brissette S, Marsh DC, Lauzon P, Guh D, Anis A, Schechter MT: Diacetylmorphine versus methadone for the treatment of opioid addiction. The New England Journal of Medicine 2009, 361(8):777-786.  ** Oviedo-Joekes E, Nosyk B, Brissette S, Chettiar J, Schneeberger P, Marsh DC, Krausz M, Anis A, Schechter MT: The North American Opiate Medication Initiative (NAOMI): Profile of Participants in North America’s First Trial of Heroin-Assisted Treatment. Journal of Urban Health : Bulletin of the New York Academy of Medicine 2008, 85(6):812-825.  ** Oviedo-Joekes E, Nosyk B, Marsh DC, Guh D, Brissette S, Gartry C, Krausz M, Anis A, Schechter MT: Scientific and political challenges in North America's first randomized controlled trial of heroin-assisted treatment for severe heroin addiction: rationale and design of the NAOMI study. Clinical trials (London, England) 2009, 6(3):261-271. |
| 1. Palter VN, Orzech N, Reznick RK, Grantcharov TP: Validation of a structured training and assessment curriculum for technical skill acquisition in minimally invasive surgery: a randomized controlled trial. Annals of Surgery 2013, 257(2):224-230. |
| 1. Patel D, Couroux P, Hickey P, Salapatek AM, Laidler P, Larche M, Hafner RP: Fel d 1-derived peptide antigen desensitization shows a persistent treatment effect 1 year after the start of dosing: a randomized, placebo-controlled study. The Journal of Allergy and Clinical Immunology 2013, 131(1):103-109 e101-107. |
| 1. Patterson ML, Moniruzzaman A, Somers JM: Community Participation and Belonging Among Formerly Homeless Adults with Mental Illness After 12 months of Housing First in Vancouver, British Columbia: A Randomized Controlled Trial. Community Mental Health Journal 2014, 50(5):604-611. |
| 1. Pelletier CA, de Zepetnek JT, MacDonald M, Hicks AL: Implementation of the Physical Activity Guidelines for Adults with Spinal Cord Injury: Effects on Aerobic Capacity and Muscle Strength. INCORPORATING PHYSICAL ACTIVITY INTO THE REHABILITATION PROCESS AFTER SPINAL CORD INJURY 2013:88. |
| 1. Perlas A, Mitsakakis N, Liu L, Cino M, Haldipur N, Davis L, Cubillos J, Chan V: Validation of a mathematical model for ultrasound assessment of gastric volume by gastroscopic examination. Anesthesia and Analgesia 2013, 116(2):357-363. |
| 1. Pollock N, Sharma N, Christenson C, Law M, Gorter JW, Darrah J: Change in parent-identified goals in young children with cerebral palsy receiving a context-focused intervention: associations with child, goal and intervention factors. Physical & Occupational Therapy in Pediatrics 2014, 34(1):62-74.   ** Law MC, Darrah J, Pollock N, Wilson B, Russell DJ, Walter SD, Rosenbaum P, Galuppi B: Focus on function: a cluster, randomized controlled trial comparing child- versus context-focused intervention for young children with cerebral palsy. Developmental Medicine and Child Neurology 2011, 53(7):621-629. |
| 1. Rabheru K, Wiens A, Ramprasad B, Bourgon L, Antochi R, Hamstra SJ: Comparison of traditional didactic seminar to high-fidelity simulation for teaching electroconvulsive therapy technique to psychiatry trainees. The Journal of ECT 2013, 29(4):291-296. |
| 1. Rosedale R, Rastogi R, May S, Chesworth BM, Filice F, Willis S, Howard J, Naudie D, Robbins SM: Efficacy of exercise intervention as determined by the McKenzie System of Mechanical Diagnosis and Therapy for knee osteoarthritis: a randomized controlled trial. The Journal of Orthopaedic and Sports Physical Therapy 2014, 44(3):173-181, A171-176. |
| 1. Rotenberg BW, Wickens B, Parnes J: Intraoperative ice pack application for uvulopalatoplasty pain reduction: a randomized controlled trial. The Laryngoscope 2013, 123(2):533-536. |
| 1. Ruzicka M, Floras JS, McReynolds AJ, Coletta E, Haddad H, Davies R, Leenen FH: Do high doses of AT(1)-receptor blockers attenuate central sympathetic outflow in humans with chronic heart failure? Clinical Science (London, England : 1979) 2013, 124(9):589-595. |
| 1. Scheifele DW, McNeil SA, Ward BJ, Dionne M, Cooper C, Coleman B, Loeb M, Rubinstein E, McElhaney J, Hatchette T et al: Safety, immunogenicity, and tolerability of three influenza vaccines in older adults: results of a randomized, controlled comparison. Human Vaccines & Immunotherapeutics 2013, 9(11):2460-2473. |
| 1. Schulman S, Kearon C, Kakkar AK, Schellong S, Eriksson H, Baanstra D, Kvamme AM, Friedman J, Mismetti P, Goldhaber SZ: Extended use of dabigatran, warfarin, or placebo in venous thromboembolism. The New England Journal of Medicine 2013, 368(8):709-718. |
| 1. Siddiqui NT, Fischer H, Guerina L, Friedman Z: Effect of a preoperative gabapentin on postoperative analgesia in patients with inflammatory bowel disease following major bowel surgery: a randomized, placebo-controlled trial. Pain Practice : the Official Journal of World Institute of Pain 2014, 14(2):132-139. |
| 1. Stacey D, Hawker G, Dervin G, Tugwell P, Boland L, Pomey MP, O'Connor AM, Taljaard M: Decision aid for patients considering total knee arthroplasty with preference report for surgeons: a pilot randomized controlled trial. BMC Musculoskeletal Disorders 2014, 15:54. |
| 1. Suman-Horduna I, Roy D, Frasure-Smith N, Talajic M, Lesperance F, Blondeau L, Dorian P, Khairy P: Quality of life and functional capacity in patients with atrial fibrillation and congestive heart failure. Journal of the American College of Cardiology 2013, 61(4):455-460. |
| 1. Sydor DT, Bould MD, Naik VN, Burjorjee J, Arzola C, Hayter M, Friedman Z: Challenging authority during a life-threatening crisis: the effect of operating theatre hierarchy. Br J Anaesth 2013, 110(3):463-471. |
| 1. Taddio A, Smart S, Sheedy M, Yoon EW, Vyas C, Parikh C, Pillai Riddell R, Shah V: Impact of prenatal education on maternal utilization of analgesic interventions at future infant vaccinations: a cluster randomized trial. Pain 2014, 155(7):1288-1292. |
| 1. Tam AK, Ilodigwe D, Li Z, Schweizer TA, Macdonald RL: Global cerebral atrophy after subarachnoid hemorrhage: a possible marker of acute brain injury and assessment of its impact on outcome. Acta Neurochirurgica Supplement 2013, 115:17-21.   ** Macdonald RL, Kassell NF, Mayer S, Ruefenacht D, Schmiedek P, Weidauer S, Frey A, Roux S, Pasqualin A, Investigators C-: Clazosentan to overcome neurological ischemia and infarction occurring after subarachnoid hemorrhage (CONSCIOUS-1) randomized, double-blind, placebo-controlled Phase 2 dose-finding trial. Stroke; a Journal of Cerebral Circulation 2008, 39(11):3015-3021. |
| 1. Taylor AM, Peck M, Launcelott S, Hung OR, Law JA, MacQuarrie K, McKeen D, George RB, Ngan J: The McGrath(R) Series 5 videolaryngoscope vs the Macintosh laryngoscope: a randomised, controlled trial in patients with a simulated difficult airway. Anaesthesia 2013, 68(2):142-147. |
| 1. Thibault B, Harel F, Ducharme A, White M, Ellenbogen KA, Frasure-Smith N, Roy D, Philippon F, Dorian P, Talajic M et al: Cardiac resynchronization therapy in patients with heart failure and a QRS complex <120 milliseconds: the Evaluation of Resynchronization Therapy for Heart Failure (LESSER-EARTH) trial. Circulation 2013, 127(8):873-881. |
| 1. Turpie AG, Hull RD, Schellong SM, Tapson VF, Monreal M, Samama MM, Chen M, Yusen RD: Venous thromboembolism risk in ischemic stroke patients receiving extended-duration enoxaparin prophylaxis: results from the EXCLAIM study. Stroke; a Journal of Cerebral Circulation 2013, 44(1):249-251.   ** Hull RD, Schellong SM, Tapson VF, Monreal M, Samama MM, Nicol P, Vicaut E, Turpie AG, Yusen RD: Extended-duration venous thromboembolism prophylaxis in acutely ill medical patients with recently reduced mobility: a randomized trial. Annals of Internal Medicine 2010, 153(1):8-18. |
| 1. Walker LM, Hampton AJ, Wassersug RJ, Thomas BC, Robinson JW: Androgen Deprivation Therapy and maintenance of intimacy: a randomized controlled pilot study of an educational intervention for patients and their partners. Contemporary Clinical Trials 2013, 34(2):227-231. |
| 1. Whelan DB, Litchfield R, Wambolt E, Dainty KN: External rotation immobilization for primary shoulder dislocation: a randomized controlled trial. Clinical Orthopaedics and Related Research 2014, 472(8):2380-2386. |
| 1. Wong DT, Tam AD, Mehta V, Raveendran R, Riad W, Chung FF: New supraglottic airway with built-in pressure indicator decreases postoperative pharyngolaryngeal symptoms: a randomized controlled trial. Canadian Journal of Anesthesia, 2013, 60(12):1197-1203. |
